# New Appliances and Things Medical

**Published:** 1901-09-07

**Authors:** 


### NEW APPLIANCES AND THINGS MEDICAL.

[We shall be glad to receive, at our Office, 28 & 29 Southampton Street, Strand, London, W.C., from the manufacturers, specimens of all new preparations and appliances which may be brought out from time to time.]

### UNSWEETENED CONDENSED MILK "EDELWEISS" BRAND.

SWISS MILK SOCIETY. LONDON AGENTS: GILBERTSON AND SONS, LTD., 11 ST. ANDREW STREET AND SHOE LANE, HOLBORN CIRCUS, LONDON, E.C.

We have examined a sample of the above unsweetened condensed milk, and find it perfectly sterile, and when employed according to the directions on the label, namely, by piercing two holes in the top and pouring out the contents, it will keep sweet quite as long as under ordinary circumstances is likely to be necessary. This is a most important point, because there is a popular belief that the addition of a large quantity of sugar is necessary in order that milk or any other preserved article of food may keep unfermented for any considerable period of time. It is claimed for this milk that it is condensed and tinned under the most careful bacteriological supervision, and that the cows which supply the milk are pasture fed for the greater part of the year. This, again, is a feature which must add to the value of the "Edelweiss" brand of milk, because cows, like human beings, are in a very much healthier condition when living in the open, than when subjected to the confinement necessitated by living day and night in a stable or cowshed. This condensed milk when diluted with an equal part of water contains the ordinary elements of cows' milk in the same proportions as is the case with a good sample of fresh dairy milk. Although it cannot be claimed that when thus prepared this milk is entirely free from the flavour which seems inseparable from all varieties of boiled milk, nevertheless the "Edelweiss" brand tastes as little condensed as any condensed milk with which we are acquainted. Owing to the freedom from added sugar it will be less distasteful than most kinds of condensed milk. In the opinion of many, for ordinary domestic use, and for invalids and infants, it has a very obvious and decided advantage.

### PLASTER OF PARIS BANDAGE.

A. DE S. DALMAS AND CO., LEICESTER.

One of the chief disadvantages associated with the use of plaster of Paris bandages is that occasionally, owing to the process of keeping under certain atmospheric conditions they do not "set" properly when applied to a limb or other, portion of the body. These new bandages appear free from this objection as far as our observations have been conducted, and we commend them to the notice of surgeons and hospital authorities.

